# Food Addiction

**DOI:** 10.3390/brainsci14100952

**Published:** 2024-09-24

**Authors:** Haley Krupa, Ashley N. Gearhardt, Anne Lewandowski, Nicole M. Avena

**Affiliations:** 1 Marian Regional Medical Center, Santa Maria, CA 93454, USA; haleykrupa@gmail.com; 2 Department of Psychology, University of Michigan, Ann Arbor, MI 48109, USA; agearhar@umich.edu; 3 Columbia College, Columbia University, New York, NY 10027, USA; apl2156@columbia.edu; 4 Icahn School of Medicine at Mount Sinai, New York, NY 10021, USA

**Keywords:** addictive, behavior, binge eating, clinical treatment, food addiction, motivation, palatable food, pharmacotherapy, neural reward pathways

## Abstract

In this review, we aim to draw a connection between drug addiction and overconsumption of highly palatable food (OHPF) by discussing common behaviors and neurochemical pathways shared by these two states. OHPF can stimulate reward pathways in the brain that parallel those triggered by drug use, increasing the risk of dependency. Behavioral similarities between food and drug addiction can be addressed by tracking their stages: loss of control when eating (bingeing), withdrawal, craving, sensitization, and cross-sensitization. The brain adapts to addiction by way of the mesolimbic dopamine system, endogenous opioids and receptors, acetylcholine and dopamine balance, and adaptations of serotonin in neuroanatomy. Studies from the current literature are reviewed to determine how various neurological chemicals contribute to the reinforcement of drug addiction and OHPF. Finally, protocols for treating food addiction are discussed, including both clinical and pharmacological modalities. There is consistent evidence that OHPF changes brain chemistry and leads to addiction in similar ways to drugs. However, more long-term research is needed on food addiction, binge eating, and their neurobiological effects.

## 1. Introduction

There is a point at which, in some cases, pleasurable behavior can turn into an addiction. Addictive drugs are a classic example of this phenomenon. One normally initiates drug use (e.g., alcohol, nicotine, or heroin) because of the euphoric and desirable feeling that is derived from it. There are a variety of underlying factors that belie pleasure-seeking drug use, like utilizing self-soothing behaviors to avoid a source of angst in a person’s life. The beginning stages of drug use are, therefore, associated with procuring pleasure. However, as an addiction to the drug develops, one’s initial pleasure-seeking motive is thought to transition to sensitized motivational urges in response to cues (i.e., “wanting”) and/or to alleviate feelings of withdrawal or dysphoria that are found in response to repeated use of the drug [1].

A discussion of the hedonic aspects of eating raises the question of whether the same biological mechanisms are at play when a person is overeating food, specifically, foods that have been refined or processed from their naturally occurring form. It is necessary to eat food for survival, and there are in-born biochemical processes that have evolved to reinforce feeding behavior to avoid starvation. Likewise, other “natural” behaviors, like sexual activity, serve as powerful reinforcers because they promote the continuation of our species. Some of our ancestors were only able to survive and reproduce if they spent most of their time hunting for food and then engaging in opportunistic bingeing due to a lack of storage methods and uncertainty about the source of their next meal. Individuals who experienced more pleasure in response to caloric foods were better at identifying caloric food cues. Thus, these individuals were more motivated to seek out calorically dense foods and in turn likely better adapted to survival in an environment where famine was a threat to survival. Thus, much of human history has selected for individual differences associated with finding caloric foods more salient and reinforcing.

However, food is plentiful today in industrialized countries, and the “natural” reinforcers have changed in modern society: there are now highly processed foods with unnaturally high levels of sugar, salt, and fat. The more potent forms of these natural reinforcers may begin to have an addictive potential, and the skills that we once needed for survival may now lead to unhealthy eating patterns that affect both the brain and feeding behaviors. As a result, there may be an inborn drive to overconsume when ultra-processed food is available, and in our current environment, this may mean people will overconsume regularly [2].

The drive to ingest food is prompted, in part, by the release of chemicals in the brain that are linked with a feeling of intense pleasure and reinforcement. As described in the next section, highly processed foods (those that are high in both refined carbohydrates and fat) are especially strong reinforcers, triggering overactivity in the biological systems intended to promote food consumption for the purpose of survival. Neurochemical changes in areas of the brain associated with pleasure and reward make the topic of hedonic eating relevant to the field of psychiatry, as it is a condition that may respond well to psychiatric interventions used to treat other forms of addiction or disordered eating. 

Animal studies have shown that the over-consumption of highly processed food induces certain behavioral and reinforcing neurological effects that are like those of addictive drugs, suggesting that they are regulated, at least in part, by a common biological mechanism [3]. Similarly to the development of a drug addiction, a food addiction may manifest more readily in a subset of genetically susceptible individuals. This may set the biological basis for the development of addictive overeating. This chapter discusses the current scientific literature, which examines behavioral and neurochemical commonalities between the over-consumption of highly processed foods and drug addiction, as well as their treatment and implications for further research.

## 2. Definitions

### 2.1. Highly Processed and Ultra-Processed Food

If eating were solely controlled by homeostatic mechanisms, the majority of us would be at our ideal body weights, and eating would be considered a dull but necessary part of existence, like breathing. However, eating is often a source of pleasure and can be associated with culture and community. Interestingly, both humans and other animals are prone to eating beyond homeostatic needs if presented with highly processed food. Highly processed food is defined as food that contains refined carbohydrates (like sugar and white flour) and/or added fats [4]. Commonly consumed highly processed foods include chocolate, ice cream, pizza, and potato chips. While individuals can buy processed ingredients (like table sugar or butter) to create highly processed food (e.g., homemade cookies or fresh-baked bread), the most common source of highly processed foods are industrially created, ultra-processed versions that also contain additives such as flavor enhancers and emulsifiers. These ultra-processed foods are often created with inexpensive, but highly reinforcing industrial ingredients (e.g., high fructose corn syrup), which allow them to be affordable. Additives and packaging also increase the shelf-stability of these products, making them ready-to-eat or ready-to-heat, thus making them accessible and convenient. Finally, ultra-processed foods are often accompanied by large marketing campaigns that increase positive expectations about the products and increase exposure to related cues in the environment.

Why is highly processed food so rewarding? Genetic studies suggest that the neurocircuitry that drives the acquisition and physical storage of food evolved in congruence with food scarcity, which promoted survival in times of famine [5]. In this evolutionary milieu, highly processed foods, while calorically dense, have typically been scarce and, through time, became more neurologically rewarding. For example, minimally processed naturally occurring foods typically only include high levels of carbohydrates (like fruit) or fat (like nuts). The frequent combination of both carbohydrates and fats in high levels found in many highly processed foods is evolutionarily novel and may have a supra-additive effect on the brain reward system [6]. There is also evidence that the unique combinations of carbohydrates, fats, and sodium in highly processed foods cause them to become hyperpalatable and consumed to excess. Further, the more rapidly reinforcing substances are absorbed into the system, the more likely they are to be addictive [7]. Ultra-processed foods are often altered in a manner that reduces the integrity of the food matrix (e.g., removal of fiber and water), which increases the bioavailability of calories and the speed at which these foods can be consumed [8]. Thus, the more rapid delivery of carbohydrates and fats into the mouth, gut, and brain would also increase the addictiveness of these products. Together, these factors may activate reward-mediated neurocircuits, which evolved originally to reinforce feeding behavior that would have been advantageous to our species during times of famine. Now, with unrestricted access to these foods, this activation presents as a maladaptive desire to engage in perpetual over-consumption [9].

### 2.2. Substance Use Disorder

A substance use disorder is characterized as two (or more) of the following symptoms being present within a period of 12 months plus clinically significant impairment/distress:

(1) *tolerance* (increased amount with a corresponding decrease in effect);
(2) *withdrawal* (negative physical or psychological symptoms arising after deprivation of a particular substance);
(3) *loss of control* (instances of a greater amount taken over time and for a period of time that is longer than intended);
(4) *a persistent desire and/or repeated unsuccessful attempts to quit using the substance*;
(5) *excessive time* spent on obtaining, using, or recovering from its effects;
(6) *reduced social, recreational, or occupational, activities* due to substance use
(7) *continued use* despite knowledge of its adverse consequences;
(8) *intense cravings*;
(9) *continued use despite interpersonal problems* due to use of the substance;
(10) *use is continued despite problems fulfilling role obligations* because of substance use; and
(11) *substance is used in situations that make it physically hazardous* [10].

While precise neurochemical mechanisms of substance use disorders are appreciably studied, research on addictive eating and its neurological correlates is still developing. Animal studies have demonstrated that excessive intake of highly processed foods (or ingredients in those foods like sugar and fat) produces behavioral and neuronal alterations that are in many ways similar to those of drug addiction [3]. 

### 2.3. Food Addiction

In the medical field, the term addiction is conventionally applied to addictive drugs and highly rewarding behaviors (like gambling). However, there is growing evidence that individuals can exhibit all the diagnostic indicators for substance use disorders in cases where the substance of abuse is highly processed foods. Food addiction is the term that has typically been applied to this addictive profile of highly processed food intake. We recognize that there is still controversy surrounding the use of food addiction as a diagnostic term. Some researchers are skeptical of where food addiction will fit in when characterizing other overfeeding behaviors like binge eating disorder (BED) and bulimia nervosa (BN). We acknowledge this perspective and appreciate the discourse that the topic has stimulated. However, there has been sufficient evidence—which will be examined later in this article—that supports the potentially addictive qualities of certain foods [11]. A commonly used measure to assess food addiction is the Yale Food Addiction Scale (YFAS), which was designed to apply the diagnostic criteria for substance use disorders to the intake of highly processed foods [12]. There are validated YFAS assessments of food addiction for both adults and children. The YFAS provides two scoring options: in the first method, one can count how many symptoms of addiction are present, and in the second a “diagnosis” of food addiction based on the SUD criteria of two or more symptoms plus clinically significant distress or impairment. Like other substance addictions, food addiction is posited to be the result of three intertwining factors—(1) the addictive nature of the substance (in this case highly processed food), (2) the risk factors of the individual (e.g., familial history of addiction, depression, or impulsivity), and (3) an environment that makes the addictive substance accessible, affordable, and appealing [13]. Recent meta-analyses have found that 14% of adults and 12% of children meet the threshold for food addiction [14,15]. For adults, this is on par with the levels of addiction to other substances that are legal, easily accessible, and addictive (like alcohol and tobacco), but the estimated prevalence for children is much higher than with other addictive substances (given that children are typically protected from other addictive substances).

While food addiction is higher in individuals with overweight and obesity, only a subset of individuals in these weight classes meet the criteria for food addiction (19–28%) [16]. Additionally, individuals who present as normal weight or even underweight can meet the food addiction criteria. Thus, an individual’s body weight should not be used as an indicator of food addiction. Instead, the behavioral criteria used to diagnose all substance use disorders should be employed (as with the YFAS). There is some overlap between food addiction and eating disorders, particularly binge-type eating disorders marked by a compulsive lack of control over intake. Evidence suggests that individuals with an eating disorder who meet the food addiction threshold exhibit a more severe psychopathological profile across a number of domains (e.g., depression, binge frequency, and neuroticism) [16]. However, food addiction should not just be considered a severe sub-type of disordered eating, as the majority of individuals who meet for food addiction do not appear to meet the diagnostic criteria for an existent eating disorder. Thus, food addiction is related to, but distinct from, obesity and eating disorders [16].

## 3. Animal Studies Relevant to Food Addiction

There are numerous motivations for binge-eating. They often involve emotional and cultural factors that are not easy to model using laboratory animals. Yet, animal models of binge-eating have proven key to bettering our understanding of the neurochemical and physiological basis of this behavior in humans. Multiple animal models of binge-eating have been developed and hold significant value, as each model has a unique relationship to human behavior; for example, some explore stress-induced eating and binge-eating of high-fat foods [17,18,19]. While chronic binge behavior is not solely dependent upon the kind of food consumed, we know that the mental state of the individual (e.g., if they are tired, anxious, depressed, or irritable) and the extent of caloric restriction also contribute to how the individual will interact with highly processed food [18]. We will focus in this paper on sugar-based bingeing since, under typical conditions, both rodents and humans have a positive and pleasurable reaction in response to the taste of sugar. Added sugars are ubiquitous in our present society in the form of highly processed food, which includes most pre-packaged food items, condiments, sugar-sweetened beverages, and more. The increase in consumption of added sugar is linked to the abrupt and continued rise in the rates of obesity [20].

Certain pathological patterns of food intake, like consistent binge-eating, overeating, emotional eating, and eating due to stress, reinforce overeating and induce patterns of behavior in striking resemblance to those seen in substance-use disorders. Consequently, there are three indicators of addiction that are demonstrated using animal models: loss of control (often indicated by bingeing), withdrawal, and craving [21]. In addition, it is thought that behavioral sensitization underlies some aspects of food addiction. Behavioral sensitization refers to a repeated process of administering an intermittent stimulant that produces a progressively greater behavioral response with each exposure [21,22].

In the next sections of this paper, empirical data will be presented that suggests addictive-like behavioral and brain changes using animal models of binge-eating. These data have been summarized in previous papers and reports [3].

### 3.1. Bingeing

Bingeing, which is defined as an escalation of sugar intake (in this context) during a narrow time frame, is usually preceded by a period of forced or voluntary restriction [3]. While some addictive patterns of intake do not include binges (e.g., steady and consistent intake of cigarettes throughout the day), binges are common across many addictive substances (e.g., alcohol and cocaine) and they are closely linked to the key addiction indicator of loss of control over consumption. The particular sugar-binge model in rodents outlined in the Avena et al. paper [3] helps elucidate the behavioral characteristics of binge behavior. In this model, rats are kept on a schedule of 12 h food deprivation, followed by 12 h access to a 10% sucrose (comparable to the sugar concentration typically found in beverages like soft drinks) and rodent chow daily [3]. In response to several days following this schedule, the rats begin to increase their intake and binge on sugar during the first hour of access [23]. Additionally, their feeding patterns begin to change, and these rats begin to eat larger sugar meals throughout the period of access when compared to control rats with unrestricted access to both the sugar to drink and chow (as well as water, which is always freely available to all rats in these studies). These control animals ingest an amount comparable to animals in the binge-eating condition, but their intake is spread over the entire 24 h of the day, and they do not show evidence of binge eating.

It is evident that current research supports the hypothesis that animals tend to selectively binge on highly palatable foods (such as sugar), suggesting this behavior could be driven by hedonic as opposed to metabolic mechanisms. The incidence in which animals tend to exhibit bingeing behavior is when there is some type of time restriction in place, as the 12 h food-deprivation paradigm suggests. In the de Sa Nogueira et al. paper, researchers evaluated neuroadaptations associated with sucrose-induced bingeing behavior in adult male rodents. This yielded the conclusion that the endocannabinoid system may provide a link between responses to the ingestion of palatable food and addictive behaviors, especially since cannabinoid mechanisms are known to be linked to drug addiction [24]. In this study, groups were provided with intermittent (12 h) or continuous (24 h) access to a solution that was 10% sucrose, and food, for 28 days. Only the 12 h access group displayed excessive sucrose intake within a discrete time period (i.e., binge eating) [24]. Furthermore, this group also exhibited changes in endocannabinoid system transcripts and endocannabinoid levels in reward-related regions of the brain. This means the more bingeing behavior the rodents exhibited, the more reward systems were engaged in accordance with the behavior. Though research is limited when it comes to understanding the role that the endocannabinoid system specifically has in binge-eating behaviors, increasing our understanding regarding the neurochemical alterations associated with binge eating puts us one step closer to determining what underlying mechanisms are at play in the brain. In addition to the Avena et al. study, other experiments show that sugar is a more influential reinforcer of binge behavior than fat or salt, and intake of sugar increases over time during repeated bouts of exposure [25]. Together, these studies demonstrate that sugar plays a key role in binge behavior.

### 3.2. Withdrawal

Withdrawal becomes apparent in animals when the substance is removed or is chemically blocked. The withdrawal syndrome includes aversive physical, cognitive, and affective symptoms that emerge following the removal of that addictive substance [26]. In opiate-dependent animals, withdrawal has well-defined and clear behavioral signs, such as anxiety (using an open-arm maze), a reduction in body temperature, aggressive behavior, dysphoria, and behavioral depression [25,27].

Behavioral characteristics of withdrawal from food have largely been studied in rats that have previously been bingeing on sugar. In one study, rats were administered naloxone, an opioid-receptor antagonist, and began to experience the somatic indicators of withdrawal, such as headshakes, teeth chattering, and forepaw tremors [28]. Food deprivation can also precipitate opiate-like signs of withdrawal, as observed in rats when food was removed for 24 h [3,28]. Therefore, these data strongly suggest that bingeing on a sugar solution triggers the release of opioids in the brain, leading to neural adaptations that manifest as dependency. Other researchers have obtained findings that support this conclusion using other models of sugar bingeing. For example, anxiety signs have been found in rats with restricted access to a diet that is high in sucrose [29]. Just the removal of sugar from the rodent’s cage can lead to a drop in body temperature [30]. Additionally, signs of aggression have been observed in cases of withdrawal from a diet that includes restricted, intermittent access to sugar [31].

### 3.3. Craving

Craving often occurs when motivation is enhanced, typically following a period of abstinence, to procure an abused substance [1]. Following a period of use and subsequent forced abstinence, animals will take more of a self-administered addictive drug that becomes available again than what they took prior to abstinence [32]. Animals may also often be persistent in operant responding despite the removal of reward (known as “resistance to response extinction”), and over time they may increase their responses for cues that were previously associated with the substance of abuse [33,34,35]. This increased motivation to obtain an addictive substance mimics behavior observed in humans, and it may help predict whether there is a likelihood of a relapse.

When applying the behavioral signs of craving in laboratory animals to food, sugar-bingeing rats show indications of enhanced motivation to get sucrose: in a test following two weeks of sugar abstinence, the rats lever-pressed for 23% more sugar than their initial quantity of sugar before abstinence [21]. However, a control group with prior half-hour access to sugar each day followed by two weeks of sugar abstinence did not show this behavior. This implies that sugar has a strong motivational impact that persists through prolonged periods of abstinence, but only in the context of prior intermittent access to sugar (i.e., 12 h access, described above) [9].

Additionally, as with addictive drugs, motivated behavior to procure sugar appears positively correlated with the length of abstinence. The seeking of sucrose seems to increase in rats that were previously maintained on an intermittent sugar access schedule after 10 days of abstinence. This behavior is even more pronounced following a period of 30 days of abstinence from sugar. This suggests a gradual development of long-term changes in the neural circuitry associated with motivation, caused by sugar self-administration and subsequent abstinence [36].

### 3.4. Sensitization and Cross-Sensitization to Psychostimulant Drugs and Alcohol

Sensitization is defined as increased responsiveness to a repeated stimulus. It is the opposite of tolerance, which is a decrease in responsiveness, in which case a stronger stimulus is required to have the same effect. Both contribute to, and are exacerbated by, binge behavior [3]. Various processes within brain systems can simultaneously lead to both sensitization (increased dopamine release) and tolerance (decreased dopamine reception). However, tolerance mechanisms are resolved within days after ceasing drug use, yet neural sensitization may last for years. This theory helps explain why a recovered addict may relapse back into addiction, even after years of sobriety, particularly with the expectation that no pleasure will be gained from a momentary relapse [37]. Similarly, cross-sensitization, where sensitization to one drug increases an animal’s susceptibility to the effects of another drug, has been demonstrated across many studies [38,39]. This “gateway effect” leads to a subsequent increase in another drug or substance [40,41,42,43,44,45].

Intriguingly, rats previously exposed to sugar-bingeing conditions have been shown to become behaviorally sensitized to the stimulant amphetamine. These animals are significantly more reactive to a low challenge-dose of amphetamine compared to naïve animals [46]. This behavior was noted after eight days of sugar abstinence. The amphetamine had little effect on any control groups (non-bingeing sugar groups, chow, etc.). On the other hand, rats that were sensitized to amphetamine show behavioral cross-sensitization to a small sugar-based meal [46]. Similar findings have been reported by other laboratories with intermittent access to sucrose cross-sensitizing rats to cocaine [47] and quinpirole, a dopamine agonist [48]. Collectively, these data bolster the idea that the dopaminergic system becomes sensitized by intermittent access to sugar, as evidenced by cross-sensitization to various dopaminergic drugs. This is significant because increased mesolimbic dopaminergic activity contributes to the behavioral effects of both sensitization and cross-sensitization [49].

When rats binge on sugar and then abstain, they have an increased intake of alcohol (9%) [50], suggesting that a limited, intermittent intake of sugar serves as a gateway to the use of alcohol. Other studies have shown animals that prefer the taste of saccharin learn to self-administer cocaine more readily than usual [51].

The behavioral parallels between drug use and hedonic overeating of highly processed food spur the question of whether highly processed food causes molecular adaptations that further promote consumption. The studies discussed in this paper suggest that this is indeed the case. Highly processed diets frequently cause adaptations in an individual’s neurobiology, permuting the drive to overeat highly processed food away from voluntary control and into compulsivity.

## 4. Neurochemical Commonalities between Drug Self-Administration and Hedonic Eating

The evidence discussed above suggests that sugar bingeing can produce behaviors that are similar to those seen in drug-dependent rats. Similarly, laboratory animal studies have been instrumental in advancing our understanding of the neurochemical effects of highly processed food and how these effects parallel those of addictive drugs. Evidence supports the hypothesis that neural systems evolved to motivate and reinforce foraging and food intake, as well as regulate drug-seeking behavior and abuse; therefore, the neurocircuitry underlying hedonic eating and drug addiction has many similarities [3]. This section discusses several neurotransmitter systems that may result in, or perpetuate, hedonic compulsive eating and are also similarly affected by drug abuse: the mesolimbic dopamine system, opioid receptors, orexin, acetylcholine, and serotonin.

Addictive drugs typically increase the signaling of dopamine from nerve endings that originate in the ventral tegmental area (VTA) to neuronal projections in the nucleus accumbens (NAc). The subsequent dopamine spike is believed to occur through direct activation of dopaminergic neurons (e.g., nicotine and stimulants), or via an indirect way through inhibition of gamma-aminobutyric acid (GABA)-ergic interneurons in the VTA (e.g., alcohol and opiates). A third mediator for drug-induced activation of dopamine neurons in the VTA is the neurotransmitter orexin. When this transmitter is released by lateral hypothalamic neurons, it broadly innervates various structures in the brain, including the VTA [12]. Similar neuroadaptations occur in regions of the limbic system after exposure to both food and drugs. These adaptations change the motivation to obtain these substances, as will be explored in detail in the following sections.

### 4.1. Mesolimbic Dopamine System

Dopamine, a neurotransmitter involved in reward and motivation, is released from neurons in the VTA and the NAc when pleasurable external stimuli are encountered. The mesolimbic dopamine system reinforces natural behaviors such as eating, sexual behavior, and socializing, but it is also stimulated by recreationally abused drugs [52]. The activation of this system by both food and drug intake suggests that a common neural mechanism may underlie the reinforcing value of both substances.

Addictive drugs can alter dopamine receptors (primarily D_1_, D_2_, and D_3_ receptors) and dopamine release in the mesolimbic brain. Drugs like cocaine upregulate D_1_ receptors [53] and increase D_1_ receptor binding [54,55], as well as increase D_3_ receptor messenger RNA (mRNA) [56], but also lower the D_2_ receptor density [57] and decrease D_2_ receptor mRNA in the NAc of laboratory animals [58,59,60]. Clinical studies also reveal that D_2_ receptors are downregulated in individuals who are addicted to cocaine [61,62,63]. Similarly, sugar-bingeing laboratory animals exhibit an increase in D_1_ receptor binding in the NAc [23], a decrease in D_2_ receptor binding in the striatum and NAc [23,64], a decrease in D_2_ receptor mRNA in the NAc [65], and an increase in D_3_ receptor mRNA in the NAc and caudate-putamen [65].

With regard to extracellular dopamine levels, the repeated increase in extracellular dopamine upon recurring exposures is a hallmark of addictive drugs [66]. Highly processed food consumed in a binge-type manner continuously releases dopamine in the NAc, resembling a pattern comparable to that seen in addictive drugs, which do not show a blunted release in the NAc upon repeated exposure [9,24,67]. In contrast, the dopamine response to bland or “normal” food fades out after repeated exposure [68].

“Priming”, in this context, occurs when a stimulus/stressor reduces extracellular dopamine levels in the NAc, subsequently exacerbating the potency of a substance (e.g., drugs or food). This phenomenon is well documented in addictive drugs, in which abstinent drug users are primed to desire or relapse with small quantities of a respective drug [69]. With highly processed food intake, energy deprivation primes rats to binge-eat because the accompanying dopamine surge within the NAc when food is reintroduced is sustained, reinforcing the reward of the highly processed foods [24,70]. Stress is another primer for binge-eating and can trigger binge-eating highly processed and less processed foods, though less potently than a history of dieting [9].

Moreover, in the model in which laboratory animals have free access to rodent chow and water ad libitum but are given sporadic, time-limited access to highly processed food to simulate binge conditions, rats consume significantly more highly processed food than the control group. This behavior stems from the repeated release of dopamine, which exacerbates the dopaminergic mechanisms involved in sustaining bingeing behaviors [71]. Together, these studies suggest that binge episodes disrupt dopamine signaling, promoting the bingeing and addictive behaviors.

### 4.2. Endogenous Opioids and Receptors

The opioid system works in concert with the dopaminergic system in both feeding and reward, and as such, is affected by hedonic overeating in a manner similar to what is seen with addictive drugs. Animal studies show that chronic access to cocaine and morphine can lead to upregulation of mu-opioid-receptor binding in the caudate-putamen, NAc, and cingulate cortex [55,72,73]. Moreover, repeated injections of morphine decrease enkephalin (an endogenous opioid) mRNA in the striatum and NAc [58,60,74]. Using brain-imaging in individuals who are dependent on cocaine, researchers have observed similar changes [75].

Ingestion of highly processed foods increases the binding of endogenous opioid-receptors in the NAc [76], and significantly decreases enkephalin mRNA in the NAc in laboratory animals [65,77]. Additionally, a history of binge-eating highly processed food (especially repeated periods of high sugar intake) may result in opioid-receptor super-sensitivity in the shell of the NAc, hippocampus, cingulate, and locus coeruleus [23]. Highly processed food stimulates opioid release in the hypothalamus, and that may explain why, in food-deprived, stressed rats, a minimal morsel of highly processed food unleashes binge-eating of bland chow, an effect that does not occur without the highly processed food trigger [18]. These studies imply that “hedonic” binge-type eating behavior may be mediated, in part, by opioid-receptor super-sensitivity, which is perhaps the result of repeated endogenous opioid release following highly processed food intake and is analogous to opiate addiction.

### 4.3. Orexin

The lateral hypothalamus (LH) is a fundamental area that bridges homeostatic and hedonic eating. Orexins, synthesized exclusively in the hypothalamus, are associated with feeding behavior—specifically, stimulating food consumption (in fact, “orexin” means “appetite”) [78]. Studies have explored the role of orexin in food and addictive drugs. Orexin has been implicated in drug abuse due to the similar neural circuitry involved in the rewarding aspects of both food and drugs [79], although the specific mechanisms for behavior remain unclear (though action sites include such reward-associated areas as the VTA and NAc) [80].

The stimulation of orexin neurons in the LH is linked with the strength of preference for cues that are predictive of drug and food reward. For example, in one study, researchers ascertained rats’ preference for morphine, cocaine, food, or no reward, and then gave rats the option to seek their corresponding preferred reward in a reward chamber, or to enter an empty chamber. Only the rats that were conditioned and showed a preference for the food or drug reward–paired chamber had increased Fos (a marker of neuronal stimulation) in LH orexin cells; rats conditioned to prefer a “novel object” reward displayed preference behavior but exhibited no enhanced orexins [81].

Interesting findings have been made with orexin regarding drug cessation. Following protracted abstinence from morphine, rats showed a proclivity for drug reward over food reward, along with alterations in Fos activation in orexins neurons within the LH [82]. A recently discovered role for LH orexin neurons within the context of food and drug reward-seeking concerns reinstatement. Chemical activation of orexin in the LH recovers extinguished drug-seeking behavior that was previously blocked by an orexin-A antagonist. Additionally, orexin-A peptide directly administered into the VTA reinstates drug-seeking [81].

In addition to orexin’s involvement in drug addiction, a neural connection between hypothalamic orexin and the NAc may modulate the rewarding aspects of highly processed food [83]. Cason et al. found that orexin signaling in the VTA stimulates intake of a high-fat diet even in rats that are sated, suggesting that a pathway linking the LH and NAc induces reward-mediated food intake in sated rats [80]. Additionally, mice that lack sweet taste receptors are nonetheless still able to develop a preference for sucrose solutions, probably because the orexin neurons that are activated upon feeding directly stimulate VTA dopamine neurons [12]. Indeed, it is hypothesized that dysregulation of the orexin system in the brain may contribute to the hedonic overeating that leads to obesity [80].

### 4.4. Acetylcholine and the Dopamine–Acetylcholine Balance

Accumbens acetylcholine (ACh) normally increases during a meal and reaches a peak when feeding ceases, which is associated with satiety. Interestingly, the elevated ACh levels are blunted in underweight rats, inciting slower satiation [84]. In the sugar addiction model by Avena et al., rats bingeing on sugar have a delay in the rise of ACh, and this probably contributes to the increase in food intake [9,66]. The irregularities in other eating behaviors and drug addiction are quite intriguing. The behavioral signs associated with drug withdrawal are typically accompanied by a decrease in dopamine and an increase in ACh in the NAc. The imbalance of DA and ACh has been demonstrated during chemically induced withdrawal from several addictive drugs, including nicotine, morphine, and alcohol [85,86,87]. This neurochemical imbalance in DA and ACh during withdrawal also occurs in sugar-bingeing rats, when they are given an opioid antagonist (naloxone) to precipitate withdrawal [28] and following 36 h of food deprivation [88]. Together, sugar binges blunt ACh release, which may reduce the feelings of satiety, but withdrawal increases ACh which, coupled with reduced dopamine levels, is postulated to create not satiety, but an aversive state such as that seen during behavioral depression, drug withdrawal, and conditioned taste aversion [3].

## 5. Serotonin

Serotonin (5-hydroxytryptamine; 5-HT) is associated with reducing food intake. Within the context of drug and sugar addiction, reduced 5-HT levels are associated with both depression and compulsive behavior, two disorders that play a role in addictive behavior [82]. Similarly, laboratory animals that have undergone calorie restriction and intermittent access to highly processed food exhibit a significant (71%) reduction in 5-HT in the medial prefrontal cortex [89]. Succinctly, binge-eating may precipitate 5-HT dysregulation, thereby strengthening the addictive urge to overeat.

### Human Studies of Food Addiction

As noted above, the YFAS has been developed to operationalize food addiction in humans by using the diagnostic criteria for substance use disorders and applying them to the consumption of highly processed foods. The first version of the YFAS based on the DSM IV was released in 2009 (with an updated version based on the DSM 5 released in 2016) [12] (Table 1). In multiple populations and samples, the versions of the YFAS have strong psychometric properties in both adults and children. The YFAS typically demonstrates a one-factor structure, strong convergent validity, internal consistency, incremental validity, discriminant validity, and predictive validity [12]. The YFAS has been cited over 1000 times and is translated and validated in several different languages (e.g., Spanish, German, Korean, Chinese, and Arabic). Thus, the YFAS provides an important methodological tool to identify individuals exhibiting signs of food addiction.

Not all foods are expected to be ingested in an addictive way. When participants were asked to indicate which foods they were most likely to consume in an addictive way (as measured by the YFAS), participants reported that highly processed foods with elevated levels of both refined carbohydrates and added fats were the most problematic (e.g., ice cream, pizza, and fries). The next category of foods with high addiction risk were those high only in refined carbohydrates (e.g., sugar-sweetened beverages and gummy candy). Highly processed foods only high in fat (e.g., bacon, steak, and cheese) were rated as having a lower addictive potential than highly processed foods with refined carbohydrates. Finally, individuals reported experiencing the lowest addictive response to minimally or unprocessed foods (like apples, salmon, beans, and carrots) [90]. This is consistent with basic science that high amounts of refined carbohydrates (like sugar) and added fats are key activators of reward circuitry and likely central to the addictive nature of these foods [3]. Further, dietary studies have found that ultra-processed foods (which are commonly high in both refined carbohydrates and added fats) are consumed at higher levels by individuals who meet the food addiction criteria [91]. 

Studies show that individuals who score higher on the YFAS are more likely to have a higher body mass index (BMI), more frequent binge-eating, elevated weight-cycling, greater impulsivity, increased emotion dysregulation, and greater attentional biases for food [92]. Individuals with YFAS food addiction are also more likely to have a familial history of alcohol problems (which is a known risk factor for addictive disorders) and to exhibit a high-risk pattern of alcohol and nicotine product consumption [93]. In addition to behavioral studies, human brain-imaging studies support the idea that dysregulated eating behaviors, which include those that are observed in obesity, may share similarities with drug addiction. Those with higher food addiction scores also have been shown to have greater activation of brain regions related to motivation when anticipating highly processed food, and reduced activation of inhibitory regions in response to food intake: both characteristics like those of drug-addicted individuals who view and subsequently use a drug [94]. Food addiction based on the YFAS has also been related to differential responses to dopamine agonists and elevated scores on a composite genetic index of higher dopamine signaling [94,95]. Thus, food addiction and drug addiction are both associated with similar dysfunction in the reward system.

## 6. Discussion and Clinical Treatment

There is increasing evidence of neurochemical and behavioral commonalities between over-consumption of highly processed foods and drug addiction, as well as the need to create effective treatments to manage overeating and obesity. It is important to consider the effects that chronic access to highly processed foods can have on the reward system, and to consider this as a contributing factor to the development of medical complications that are linked to body weight and diet, as well as a potential target for treatment [96].

Interventions for this effect include the use of pharmacological treatments that reduce palatability in conjunction with behavioral therapies, which may prove beneficial in diminishing the perceived palatability of foods, thereby reducing food intake [97]. Specifically, a neurobehavioral model of treatment suggests that reward sensitivity to the highly processed food that drives hedonic overeating is usually coupled with insufficient inhibitory control. Strengthening this inhibitory control, through pharmacological or other clinical means, could lessen the effect of highly processed food on individuals who exhibit an addiction to these foods [98].

A number of new treatments for obesity targeting neural areas associated with food addiction are currently in phase II and phase III clinical trials [99]. Most of these potential treatment options target the neural pathways and neurotransmitters discussed in this chapter. Specifically, bupropion, raclopride, and antipsychotics target the dopaminergic system; naloxone, naltrexone, and nalmefene target the opioid system; baclofen and topiramate target the GABA-ergic system; and novel targets of the cannabinoid receptors [100].

In addition, there are medications for the treatment of type 2 diabetes that have become increasingly popular in the weight loss and weight maintenance space. This class of medication is known as glucagon-like peptide-1 receptor antagonists, often referred to as GLP-1 antagonists. Brand names such as Ozempic, Trulicity, and Victoza all work in the beta-cells of the pancreas to enhance glucose-induced insulin secretion. GLP-1 also appears to be a physiological regulator of appetite and food intake [100,101], explaining their role in potential weight loss. These medications come with important risks to consider, and many of these drugs have side effects, including increased risk of anxiety, obsessive-compulsive disorder, depression, seizures, confusion, suicide, or memory deficits [99]. Details of some of the aforementioned drugs and their neurochemical effects are outlined below.

### 6.1. Dopamine

Dopamine D_2_ receptors are consistently associated with food reward and consumption. Differential effects of D_2_ receptor blockade on highly processed food consumption have been reported. For example, Corwin et al. [102] found that the D_2_ receptor antagonist raclopride selectively attenuated binge-consumption of vegetable fat precipitated by limited access, while it had no effect on ad libitum ingestion of the food. In rats fed an ad libitum high-fat diet, raclopride reduced consumption of the high-fat diet at high doses, but increased consumption at lower doses [102]. In addition, the schedule of access to fat varies the effects of D_2_ receptor blockade: raclopride was less effective at reducing fat intake in non-deprived rats with intermittent access to fat as vs. rats with daily, one-hour access [103,104]. By contrast, raclopride has been shown to attenuate sucrose intake regardless of the access schedule [105].

### 6.2. Opioids

Naltrexone, an opiate-receptor antagonist, is used as a treatment for alcohol dependence and is approved by the Food and Drug Administration (FDA); however, its efficacy is debated [106]. Notwithstanding, several animal studies support the efficacy of opioid antagonists in reducing binge-like food consumption, though under variable circumstances. An opiate-receptor blockade may be more effective at reducing hedonic overeating of fat and sugar bingeing [18], which is especially evident with the use of the opiate-receptor antagonist naltrexone, which exerts its influence on reward-related areas of the brain (e.g., amygdala) rather than homeostatic-related areas (e.g., hypothalamic paraventricular nucleus) [107]. Another study suggests that, because opioids play a role in reward as well as in pleasure, naltrexone reduces short-term highly processed food intake and reduces pleasantness ratings for foods without affecting hunger [12]. An additional study concluded that treatment with naltrexone, in combination with a reduced-calorie diet and increased physical activity, was a well-tolerated and effective option for improving disordered eating behavior and promoting weight loss in obese patients with binge eating disorder [108]. Finally, fluoxetine (alone and as an adjuvant to naltrexone) effectively reduces the frequency of binge-eating in both open-label trials as well as in case studies [109].

### 6.3. Serotonin

Serotonin (5-HT) is involved with hedonic eating by inhibiting food intake. Individuals with a history of binge-eating behavior have reduced 5-HT transporter binding; however, after treatment with a selective serotonin reuptake-inhibitor (SSRI), fluoxetine, 5-HT binding increased significantly [109]. SSRIs—such as fluoxetine and citalopram—which are typically used to treat depression, have demonstrated efficacy at reducing the frequency of binge behavior but do not significantly result in weight loss. However, the serotonin and norepinephrine reuptake-inhibitor sibutramine is efficacious at reducing both short-term binge frequency and weight [110]. In animal studies, fluoxetine attenuates binge-eating of highly processed food in rats with a history of food restriction and sporadic access to highly processed food [88]. Interestingly, fluoxetine also reduces intravenous cocaine self-administration in rats [109]. Serotonin transporter binding is an adaptive mechanism that can be affected by treatment, and has been shown to reduce binge-eating in short-term studies.

### 6.4. Orexin

Orexin systems are implicated with inciting highly processed food- and drug-seeking behavior when cue-stimulated (even in the absence of reward). Researchers have observed overlap in the treatment of hedonic overeating and substance addiction with orexin antagonists (similar treatment for obesity as for alcohol-seeking) in rats [111,112]. Specifically, blocking orexin receptor-1 (OxR1) signaling can attenuate the cue-induced reinstatement of sucrose-seeking, primarily in rats that are food-restricted [79]. The results from preliminary studies have implications for humans as well: interference with the orexin system using an orexin-1 receptor antagonist affects long-term energy balance via both food intake and weight reduction [113]. Orexin systems are valid targets for the pharmacotherapy of binge-eating.

### 6.5. Ineffective Pharmacological Treatment

The effectiveness of pharmacological interventions may be contingent, to some extent, upon the macronutrient composition of a binge. For example, an intervention targeting the dopamine system was more effective during a high-fat, low-sugar meal, and less effective with higher sugar content, especially when the sugar was consumed within a short amount of time [113]. To further illustrate, drugs such as the GABA receptor agonist baclofen, the D_2_ receptor antagonist raclopride, and the opioid-receptor antagonist naltrexone, though effective in reducing intake of mixtures with low levels of sucrose but high levels of fat, are ineffective at preventing bingeing in rats when exposed to both high-fat and high-sugar mixtures [12,95]. This highlights the importance of a multidimensional treatment approach that limits both fat and sucrose concentrations in binge foods (or limits access to highly processed foods) in addition to pharmacological treatment.

To date, current treatment options for food addiction are still not well established, mainly due to the lack of studies that are controlled and contain large samples of patients. Notwithstanding, the data on serotonin and norepinephrine reuptake inhibitors and on anticonvulsants hold promise with respect to both efficacy and tolerability. There also exist promising data that suggest the possibility of regulating the desire to eat through an interference with the ghrelin and GLP-1 system [114]. However, emerging evidence aside, our current understanding of food addiction treatment is wanting; no clear treatment protocol has yet been determined for addictive eating.

### 6.6. Non-Pharmacological Treatment

Some individuals eat addictively as a way to self-medicate or to soothe negative emotional states such as loneliness, boredom, anxiety, depression, and conflict. A history of trauma and post-traumatic stress disorder symptoms is also elevated in individuals with food addiction [115,116]. Consequently, behavior modification is often a necessary and effective addition to pharmacological interventions in the treatment of food addictions [6]. Indeed, certain behavioral interventions have been useful for both food and drug addiction; namely, incentive motivation, cognitive behavioral therapy, 12-step programs, and motivational interviewing [5]. Notwithstanding the optimal support of any of these treatment methods, treatment of addictive eating may still be a laborious process, punctuated by alternating periods of relapse and recovery [12]. This is particularly true in a food environment dominated by cheap, easily accessible, and heavily marketed ultra-processed foods high in refined carbohydrates and added fats. Clinical treatment combined with pharmacological treatment may provide a helpful alternative for many individuals to achieve more significant, long-lasting changes. In general, rigorous, long-term research on optimal treatments for individuals endorsing an addiction to highly processed foods is a significant need given the high levels of psychopathology and lower quality of life associated with this profile [12,117].

## 7. Conclusions

Chronic overeating of highly processed foods can alter brain function in ways similar to addictive drugs. Long-standing neurobiological research (i.e., positron emission tomography scans and fMRI imaging) has provided insight into how drug and alcohol addiction affects brain systems. Building on these studies, which have provided addiction neuroscience models, further research with laboratory animals has rendered considerable evidence to support the theory that both addictive drugs and the consumption of highly processed foods utilize a shared pathway within the limbic system to mediate motivated behaviors. Neurologically speaking, highly processed foods can act like a traditional addictive drug, altering brain function in ways similar to drugs, particularly within the mesolimbic dopamine-reward pathway. Similarly, pharmacological interventions that extinguish drug addiction and cravings may also be effective at reducing addictive eating behavior, though more research is warranted. Despite great advancements in understanding the short-term effects of hedonic eating, there is a paucity of long-term research on food addiction and its treatment. Determining the long-term consequences of diets high in sugar, salt, and fat on the limbic system and on human behavior will provide insights into the underlying causes and treatments of food addiction. Greater knowledge of the addictive nature of highly processed foods will also inform the need for policies and legislation aimed at altering a food environment dominated by these substances.

## 8. Future Directions for Research

The study of food addiction and feeding disorders is still a relatively new field, and, like many medical phenomena, is subject to complex, intersectional factors. It would be valuable to explore the ways in which demographic and other social determinants contribute to hedonic eating in different communities. Approaching chronic overeating from a holistic perspective will ultimately lead to more effective identification and treatment of its physiological, psychological, and environmental aspects.

## Figures and Tables

**Table 1:** Application of the DSM-5 substance-use disorder criteria to food addiction.

DSM-5 Criteria for Substance-Use Disorders *	Relation to Food Addiction
**Criterion A: Impaired Control over Substance Use**	
Individual may take substance in larger amounts or over a longer period than originally intended	Unintended hyperphagia; eating despite lack of hunger; eating until feeling physically ill
Individual may express a consistent desire to reduce or regulate substance use and may report many unsuccessful efforts to do so	Dietary restraint; repeated failed attempts to limit the consumption of particular foods
Individual may spend a significant amount of time obtaining the substance, using the substance, or recovering from its effects	Going out of one’s way to obtain certain foods; eating throughout the day; feeling sluggish after overeating
Craving is manifested by an intense desire or urge for the drug	Overwhelming urge to consume certain foods; preoccupied by thoughts of food and eating
**Criterion B: Social Impairment**	
Recurrent substance use may result in failure to fulfill obligations at work, school, or home	Overeating that results in obesity can limit recreational activities and the ability to perform some aspects of one’s job or household chores
Individual may continue using substance despite having persistent or recurrent social or interpersonal problems caused or exacerbated by effects of the substance	Individuals often get into arguments with loved ones about the amount or way they are eating, akin to fighting about smoking
Important social, occupational, or recreational activities may be given up or reduced because of substance use	Professional or social situations may be avoided based on food availability (e.g., a certain food is absent or fear of overeating foods present). Also, overeating that leads to obesity can limit participation in activities
**Criterion C: Risky Use of the Substance**	
Recurrent substance use in physically hazardous situations	Bingeing on sugar despite having diabetes or another comorbidity that poses an immediate hazard to one’s health
Continued substance use despite knowing of having a persistent or recurrent physical or psychological problem that is likely to have been caused or exacerbated by the substance	Food habits are continued despite physical health concerns (i.e., diabetes, hypertension, excessive weight gain, cardiovascular disease) or psychological health concerns (i.e., depression, low self-esteem, eating disorders characterized by binge-eating)
**Criterion D: Pharmacological Criteria**	
Tolerance is signaled by requiring a markedly larger dose of the substance to feel the desired effect or a markedly reduced effect when the usual dose is consumed	Laboratory animals show escalation of highly processed food intake over time in binge paradigms. Humans also report needing greater amounts of food over time to achieve the same effect, including reducing negative emotions like sadness or increasing pleasure
Withdrawal is a syndrome that occurs when blood or tissue concentrations of a substance decline in an individual who had maintained prolonged heavy use of the substance	Opiate-like withdrawal has been observed in laboratory animal models. Humans may feel irritable, nervous, or sad; food is used to alleviate negative physical symptoms or emotional problems, and when certain foods are cut down, physical symptoms occur (e.g., headaches and fatigue)

* Severity of disorder is categorized from mild to severe depending on the number of criteria met, with two to three symptoms indicating mild severity, four to five suggesting moderate severity, and six or greater indicating a severe substance-use disorder. Whether clinically significant impairment or distress is also present is considered when making a diagnosis [10].
